# Peripheral Blood WT1 Expression Predicts Relapse in AML Patients Undergoing Allogeneic Stem Cell Transplantation

**DOI:** 10.1155/2014/123079

**Published:** 2014-08-17

**Authors:** Michele Malagola, Cristina Skert, Giuseppina Ruggeri, Alessandro Turra, Rossella Ribolla, Valeria Cancelli, Federica Cattina, Elisa Alghisi, Simona Bernardi, Simone Perucca, Andrea Di Palma, Erika Borlenghi, Chiara Pagani, Giuseppe Rossi, Luigi Caimi, Domenico Russo

**Affiliations:** ^1^Unit of Blood Disease and Stem Cell Transplantation, Department of Clinical and Experimental Sciences, University of Brescia, AO Spedali Civili di Brescia, P.le Ospedali Civili 1, 25123 Brescia, Italy; ^2^Chair of Biochemistry, Department of Molecular and Translational Medicine, Univeristy of Brescia, Laboratorio Analisi, AO Spedali Civili di Brescia, P.le Ospedali Civili 1, 25123 Brescia, Italy; ^3^Division of Hematology, AO Spedali Civili di Brescia, P.le Ospedali Civili 1, 25123 Brescia, Italy

## Abstract

To evaluate if WT1 expression may predict relapse after allo-SCT, we analyzed WT1 levels on peripheral blood (PB) and bone marrow (BM) before and after allo-SCT in 24 AML patients with WT1 overexpression at diagnosis. Five copies of WT1/ABL × 10^4^ from PB were identified as the threshold value that correlated with relapse after allo-SCT. The same correlation was not identified when WT1 expression was assessed from bone marrow (BM). Eight out of 11 (73%) patients with a pre-allo-SCT PB-WT1 ≥ 5 and 4/13 (31%) patients with a pre-allo-SCT PB-WT1 < 5 relapsed, respectively (*P* = 0.04). The incidence of relapse was higher in patients with PB-WT1 ≥ 5 measured after allo-SCT, at the 3rd (56% versus 38%; *P* = 0.43) and at the 6th month (71% versus 20%; *P* = 0.03). Patients with pretransplant PB-WT1 < 5 had significantly better 2-year OS and LFS than patients with a PB-WT1 ≥ 5 (81% versus 0% and 63% versus 20%) (*P* = 0.02). Our data suggest the usefulness of WT1 monitoring from PB to predict the relapse in allotransplanted AML patients and to modulate the intensity of conditioning and/or the posttransplant immunosuppression in an attempt to reduce the posttransplant relapse risk.

## 1. Introduction

The Wilms tumor gene (WT1), originally defined as a tumor suppressor gene, is also a gene transcription factor overexpressed in leukemic cells, where it induces inhibition of apoptosis and differentiation [1, 2]. It is highly expressed in more than 80% of acute myeloid leukemia (AML) patients, both in bone marrow (BM) and in peripheral blood (PB), and it is considered a panleukemic marker of minimal residual disease (MRD) [3, 4], used especially in those patients (about 50% of cases) who do not have a suitable specific cytogenetic or molecular marker.

Studies investigating WT1 as a marker of MRD have clearly demonstrated that its expression is low in normal bone marrow, is increased in AML patients at diagnosis, is decreased after an effective treatment, and becomes elevated again prior to clinical relapse [1, 2, 5–10]. Although monitoring the MRD with WT1 affords the opportunity to evaluate the majority of AML patients, its prognostic or predictive value is not collectively recognized and confirmed, so that many questions remain open. For example, the level of WT1 at diagnosis has not been clearly correlated with complete remission (CR), overall survival (OS), and leukemic free survival (LFS) [5, 6, 9, 10]; furthermore, the posttreatment threshold level of WT1 is still not well defined and actually the time-point of evaluation (postconsolidation rather than postinduction) and the source of leukemic cells (BM or PB) together with the lack of a single standardized WT1 assay are some of the most important points still debated [1, 2, 5–8, 10–19].

In this study, we performed a retrospective analysis to evaluate the predictive value of WT1 expression in 24 AML patients who were consecutively submitted to allogeneic stem cell transplantation (allo-SCT) between June 2009 and September 2013.

## 2. Patients and Methods

### 2.1. Patients

Twenty-four adult AML patients, aged between 18 and 65 years, consecutively allotransplanted at our Center between June 2009 and September 2013 were enrolled in this study. All these patients had a high-risk AML at diagnosis, according to the ELN criteria [20], and were allotransplanted in first or second complete remission (CR), defined according to the ELN criteria [20], after a conventional induction/consolidation treatment program. The characteristics of the patients are reported in Table 1. Briefly, the median age was 51 years (24–63); 92% of patients were transplanted in 1st CR; 67% of them received a graft from a matched unrelated donor (MUD); 46% of them received a myeloablative conditioning regimen; and 88% of patients received peripheral blood stem cells. Additional molecular markers of MRD, other than WT1, were Flt3-ITD in 4 cases (17%) and NPM-1 mutation in 6 cases (25%).

### 2.2. Assessment of WT-1 Expression

WT1 expression was measured from PB and BM samples collected before transplantation and at the 3rd and at the 6th month after allo-SCT. According to the European Leukemia Net (ELN) assay, real-time quantitative transcription polymerase chain reaction (RQ-PCR) normalized to ABL gene was used to assess WT1 expression [6]. Levels of WT1 were expressed as copies of WT1/ABL × 10^4^ [6]. All experiments were carried out in duplicate with appropriate positive and negative controls. The results showing a discrepancy >1 Ct between the two wells were excluded and repeated and the samples containing less than 10^4^ copies of ABL were evaluated as degraded and inadequate for analysis according to EAC criteria. Concerning our series, the median expression of ABL in all samples was 21431 copies (range 12617–39393). The median copies of PB-WT1 before allo-SCT, and after allo-SCT, at the 3rd month and at the 6th month, were 6.25 (range 0.35–188), 2.26 (range 1–953), and 2.4 (range 0.47–10353), respectively. Similarly, the median copies of BM-WT1 before and after allo-SCT, at the 3rd month and at the 6th month, were 30.9 (range 7.8–9804), 40.65 (range 5.8–10704), and 37.91 (range 9.9–14257), respectively.

### 2.3. Statistical Analysis

Patient's characteristics were summarized by standard descriptive statistics. The Mann-Whitney *U* test was used to compare continuous values. To estimate the cut-off point of WT1 levels for relapse rate, continuous values of WT1 from BM and PB were categorized at approximately the 25th, 50th, and 75th percentile. If the relapse rate in 2 or more adjacent categories was not substantially different, the categories were grouped together. If no clear pattern was observed, the median was taken as the cut-point. Survival distributions (overall survival—OS—and leukaemia free survival—LFS) were calculated using the Kaplan-Meier method [21]. OS was calculated from the date of transplant until the date of death (whatever the cause). Patients still alive were censored at the last follow-up. LFS was calculated from the date of transplant until the date of disease recurrence or until death, whichever occurred first. All *P* values were 2-sided and *P* < 0.05 was considered as statistically significant.

## 3. Results

The assessments of PB-WT1 and BM-WT1 levels were concomitantly performed at a median of 18 days before allo-SCT (range 15–21). By categorizing the continuous values of PB-WT1, we identified the cut-off of 5 copies/ABL × 10^4^ as the threshold value that was correlated with relapse after allo-SCT. On the contrary, we were not able to identify any threshold performing the analysis on BM-WT1 levels. The distribution of patients' characteristics according to pretransplant PB-WT1 < or ≥5 is reported in Table 1. Eleven out of 24 (46%) had a ≥5 PB-WT1/ABL × 10^4^, whereas 13/24 (54%) had a <5 WT1/ABL × 10^4^. Higher Flt3-ITD (31%), lower NPM-1 mutations (8%), and higher frequency of MUD (85%) were segregated in the group of patients with PB-WT1/ABL × 10^4^ < 5, while no differences were observed among the other characteristics.

When considering PB-WT1 level before allo-SCT, 8/11 (73%) patients with PB-WT1 ≥ 5 relapsed after a median time of 5 months (range 2–9). This parallels the 4/13 (31%) relapses observed after a median time of 6 months (range 6–12) in the group of patients with PB-WT1 level < 5 (*P* = 0.04).

Table 2 reports the outcome according to PB-WT1 levels, 3 and 6 months after allo-SCT. The incidence of relapse was higher in AML patients with PB-WT1 ≥ 5 measured at the 3rd (56% versus 38%; *P* = 0.43) and the 6th month (71% versus 20%; *P* = 0.03) after allo-SCT. Interestingly, 5/5 (100%) patients with pretransplant PB-WT1 ≥ 5 who never reduced this level at the 3rd or the 6th month after allo-SCT experienced a disease recurrence.

**Figure 1:**
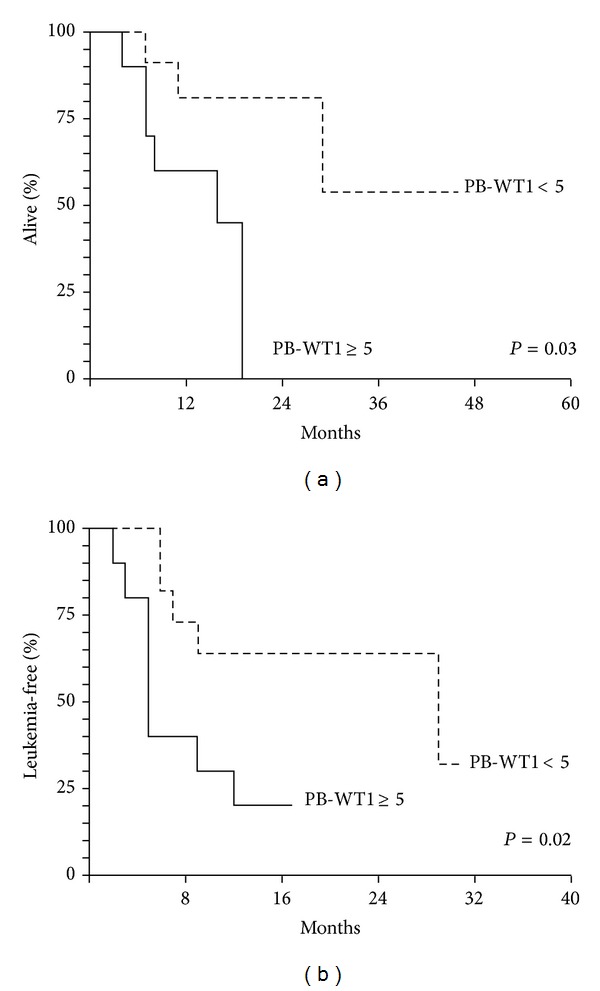
(a) OS of the 24 AML patients according to PB-WT1 level before allo-SCT. (b) LFS of the 24 AML patients according to PB-WT1 level before allo-SCT.

The median follow-up after transplantation is 12 months (range: 2–46). The OS and the LFS according to pretransplant PB-WT1 levels are reported in Figures 1(a) and 1(b). Patients with pretransplant PB-WT1 < 5 had a significantly better OS than patients with a PB-WT1 ≥ 5 at 1 year (81% (95% CI 57–100) versus 60% (95% CI 30–90)), at 2 years (81% (95% CI 57–100), versus 0%), and at 3 years (54% (95% CI 8–100) versus 0%) (*P* = 0.03) (Figure 1(a)). Similarly, the LFS of patients with pretransplant PB-WT1 < 5 at 1 year (63% (95% CI 35–92)), 2 years (63% (95% CI 35–92)), and 3 years (32% (95% CI 0–78)) was significantly longer in comparison to LFS of patients with pretransplant PB-WT1 ≥ 5 (20% (95% CI 0–45) at 1 year, 2 years, and 3 years; *P* = 0.02) (Figure 1(b)).

*Postrelapse Treatment*. Twelve (50%) out of 24 patients experienced clinical disease recurrence, which was preceded by a molecular disease recurrence in 2 cases. Nine (75%) out of the 12 relapsed patients received a salvage treatment. This consisted in Azacytidine (4 cases), Azacytidine + donor lymphocyte infusions (DLI) (2 cases), intensive chemotherapy (1 case), second allo-SCT (1 case), and DLI (1 case). Overall, 2/11 (18%) relapsed patients obtained and maintained a CR after salvage treatment (second allo-SCT in one case and intensive chemotherapy in the other) and 2/11 (18%) are alive with active disease.

## 4. Discussion

WT1 expression is helpful to monitor minimal residual disease (MRD) in 80% or more of patients with AML [5–7, 10]. However, its clinical usefulness is not well ascertained and currently WT1 is neither used for risk stratification of newly diagnosed AML patients, nor used to give different therapeutic strategies. The main limit to clinical use of WT1 is essentially due to its low reliability. This is a consequence of the lack of a single agreed standardized method of analysis and of a well defined threshold level predictive for relapse [1–18].

Although our data originate from a small unicentric group of patients, but with a relatively long follow-up (median of 12 months, range: 2–46), they suggest that WT1 expression assessed before allo-SCT may be a useful tool to identify AML patients who are at high risk of relapse. Thus, assessing WT1 expression can help to drive transplant strategy, by modulating not only the posttransplant immunosuppression but also the intensity of pretransplant conditioning regimen.

In our study, we identified a PB-WT1 ≥ 5 before transplant as the threshold level significantly correlated with higher relapse rate after allo-SCT (*P* = 0.04). On the contrary, we did not find any correlation between pre-allo-SCT BM-WT1 levels and the risk of relapse. Since WT1 expression is thought to reflect the burden of more immature leukemic cells, WT1 expression measured from PB could be more sensitive and reliable than WT1 expression measured from BM. Indeed monitoring PB samples could allow a reduction of the variability due to the harvest of BM samples and, furthermore, it may offer the advantage of easier check and more stringent evaluations over time.

Time of evaluation of WT1 expression is very important. It is known that WT1 expression is low in normal controls [6]. Therefore, it may be reasonable to think that WT1 levels in samples collected not after a single course of therapy but after a therapeutic program of intervention should be very close to normals. We measured WT1 expression before allo-SCT, after induction and at least two consolidation courses; therefore, it is not surprising that patients at low risk of relapse after allo-SCT had PB-WT1 levels very close to normals.

As we said before, the predictive value of WT1 expression before allo-SCT is clinically relevant to planning the therapeutic strategy, but monitoring WT1 levels after allo-SCT may be useful to further refine prediction of relapse. Interestingly, 5/5 (100%) patients with a PB-WT1 before allo-SCT ≥ 5 who did not reduce MRD at the 3rd month experienced a recurrence of disease in the first 6 months after allo-SCT.

In many published papers, different methods to detect MRD in AML patients (e.g., multiparameter flow cytometry, WT1 levels, molecular chimerism,…) have been compared, but none emerged as more powerful than another in predicting disease relapse [8, 12, 17, 19]. A comparison between the prognostic relevance of MRD measurement with PB-WT1 and other available markers (e.g., Flt3-ITD or NPM-1 mutation) was not an objective of our study. Nevertheless, the Flt3-ITD and NPM-1 mutation negativity checked before allo-SCT and at the 3rd and 6th month was concordant with PB-WT1 expression < 5 in all of the nonrelapsing patients.

Most of the published trials investigating WT1 expression in MRD monitoring of AML employed BM source for its assessment [3–5, 7–18]. In this respect, our study could be closely compared with the studies of the Czech Republic group [1, 2], because PB source was mainly used for WT1-MRD monitoring. The threshold level observed in our experience resulted lower and clearly different from the one reported by these authors [1, 2] and this could probably be due to the different method of data analysis. While they set the cut-off based on the median of WT1 values found in patients in permanent hematological remission [2] or adopted the ELN cut-off [1], we identified our PB-WT1 cut-off (< or ≥5) by categorizing the continuous values of WT1 at approximately the 25th, 50th, and 75th percentile. We also observed that the WT1 median values of nonrelapsing patients were lower than the median values of relapsing patients, but in our case we were not able to replicate the results of previous studies in which the mean/median WT1 values [11, 12] or other cut-offs [5, 7, 8, 10, 13–19] were used to stratify patients at different risks of relapse. The wide variability in the assessment of WT1 positivity is one of the major factors explaining the great variability of results reported in the literature [1, 2, 11, 12, 14–19]. As an example, some authors set the cut-off based on internal control whether using or not using a receiving-operating characteristic (ROC) analysis [17], while others express WT1 levels using GUS gene [13] and taking cell line K562 as calibrator [12].

In conclusion, our study makes a contribution in favor of the usefulness of monitoring PB-WT1 expression in AML patients undergoing allo-SCT. Currently, we are prospectively validating our results, but other prospective studies are warranted to confirm PB-WT1 as a reliable predictive marker for AML recurrence in the setting of allotransplanted patients. Maybe the best time to achieve a reliable WT1-MRD measure is close to transplant, at the end of the induction and consolidation treatment program. This is probably the best time to achieve a reliable measure of residual leukemic cell burden in any case. In this regard, evaluation of PB-WT1 appears to be more sensitive and advantageous than evaluation of BM-WT1.

## Figures and Tables

**Table 1:** Characteristics and relapse incidence of AML patients grouped according to PB-WT1/ABL × 10^4^ ≥ 5 or <5 before allo-SCT.

Variable	Total	PB-WT1/ABL × 10^4^ ≥ 5	PB-WT1/ABL × 10^4^ < 5	*P*
	(*n* = 24)	(*n* = 11)	(*n* = 13)	
Median age [range]	51 (24–63)	49 (42–63)	51 (24–63)	NS
Disease phase at allo-SCT				
First CR	22 (92%)	10 (91%)	12 (92%)	NS
Second	2 (8%)	1 (9%)	1 (8%)	
or subsequent remission				
Specific molecular marker				
Flt3-ITD	4 (17%)	0	4 (31%)	0.04
NPM-1 mutation	6 (25%)	5 (45%)	1 (8%)	0.03
Donor source				
Sibling	8 (33%)	6 (55%)	2 (15%)	0.04
MUD	16 (67%)	5 (45%)	11 (85%)	
Conditioning				
MAC	11 (46%)	5 (45%)	6 (46%)	NS
RIC	13 (54%)	6 (55%)	7 (54%)	
Stem cells source				
BM	3 (12%)	0	3 (23%)	NS
PB	21 (88%)	11 (100%)	10 (77%)	
Posttransplant relapse	12 (50%)	8 (73%)	4 (31%)	0.04
Median time	6	5 months	6 months	NS
(range)	(2–12)	(2–9)	(6–12)	

CR: complete remission; MUD: matched unrelated donor; UCB: umbilical cord blood; MAC: myeloablative conditioning; RIC: reduced-intensity conditioning; BM: bone marrow; PBSC: peripheral blood stem cells; NS: nonsignificant.

**Table 2:** Patients' outcome according to PB-WT1 levels (</≥5 WT1/ABL × 10^4^) evaluated before and after allo-SCT.

	PB-WT1/ABL × 10^4^ ≥ 5	PB-WT1/ABL × 10^4^ < 5	*P*
Before allo-SCT			
Number of cases	11	13	
Number of relapses	8 (73%)	4 (31%)	0.04
At 3rd month after allo-SCT			
Number of cases	9	13	
Number of relapses	5 (56%)	5 (38%)	0.43
At 6th month after allo-SCT			
Number of cases	7	10	
Number of relapses	5 (71%)	2 (20%)	0.03
